# Preface

**DOI:** 10.2174/1573403X2001240311152907

**Published:** 2024-03-11

**Authors:** Tong Liu

**Affiliations:** 1 Second Hospital of Tianjin Medical University, Tianjin Institute of Cardiology, Department of Cardiology, Tianjin 300211, People’s Republic of China

   Current Cardiology Reviews is a journal dedicated to reporting new and significant developments in cardiovascular physiology and physiopathology, as well as prevention, diagnosis, and treatment of cardiovascular diseases, which has grown over the years into a reputable international journal that continues to spotlight the latest developments in cardiology.

   This issue of Current Cardiology Reviews includes several high-quality review articles that provide insights into the novel risk factors and preventive and protective strategies for cardiovascular diseases. The development described in this issue highlights the tremendous progress that has been made in cardiology. Therefore, I would like to thank all the authors who contributed to the articles in this issue.

   I am also sincerely grateful to the associate and section editors, as well as the editorial board members, for their constant support and guidance, which have been major factors in its growth and popularity.

   I look forward to high-quality contributions across the world and hope that you enjoy reading this diverse collection of articles.

